# Long-term Neurological Outcomes in Adults with Traumatic Intracranial Hemorrhage Admitted to ICU versus Floor

**DOI:** 10.5811/westjem.2015.1.23356

**Published:** 2015-03-02

**Authors:** Daniel K. Nishijima, Joy Melnikow, Daniel J. Tancredi, Kiarash Shahlaie, Garth H. Utter, Joseph M. Galante, Nancy Rudisill, James F. Holmes

**Affiliations:** * University of California, Davis, Department of Emergency Medicine, Davis, California; † University of California, Davis, Center for Healthcare Policy and Research, Davis, California; ‡ University of California, Davis, Department of Neurological Surgery, Davis, California; § University of California, Davis, Department of Surgery, Davis, California

## Abstract

**Introduction:**

The objective of this study was to compare long-term neurological outcomes in low-risk patients with traumatic intracranial hemorrhage (tICH) admitted to the ICU (intensive care unit) versus patients admitted to the floor.

**Methods:**

This retrospective study was conducted at a Level 1 trauma center from October 1, 2008, to February 1, 2013. We defined low-risk patients as age less than 65 years, isolated head injury, normal admission mental status, and no shift or swelling on initial head CT (computed tomography). Clinical data were abstracted from a trauma registry and linked to a brain injury database. We compared the Extended Glasgow Outcome Scale (GOS-E) score at six months between patients admitted to the ICU and patients admitted to the floor. We did a risk-adjusted analysis of the influence of floor admission on a normal GOS-E.

**Results:**

We identified 151 patients; 45 (30%) were admitted to the floor and 106 (70%) to the ICU. Twenty-three (51%; 95% CI [36–66%]) patients admitted to the floor and 55 (52%; 95% CI [42–62%]) patients admitted to the ICU had a normal GOS-E. On adjusted analysis; the odds ratio for floor admission was 0.77 (95% CI [0.36–1.64]) for a normal GOS-E at six months.

**Conclusion:**

Long-term neurological outcomes in low-risk patients with tICH were not markedly different between patients admitted to the ICU and those admitted to the floor. However, we were unable to demonstrate non-inferiority on adjusted analysis. Future work aimed at a larger, prospective cohort may better evaluate the relative impacts of admission type on outcomes.

## INTRODUCTION

Each year traumatic brain injury (TBI) accounts for an estimated 1.4 million emergency department (ED) visits in the United States.^1^ Approximately 95% of patients with TBI are categorized as mild.^2^ In patients with mild TBI with intracranial hemorrhage, routine admission to the hospital for early detection of secondary brain injury is recommended.^2^ Because most of these patients do not develop hemorrhage progression or require neurosurgical intervention, significant variation in ED disposition exists, including admission to the intensive care unit (ICU), admission to the hospital floor, or ED observation.^3^–^6^ In one study, variability in ICU use among Western U.S. trauma centers ranged from 50% to 97% for patients with minor traumatic intracranial hemorrhage.^6^

We recently derived a clinical instrument that identified a subset of patients with mild TBI with intracranial hemorrhage who likely do not require ICU admission.^7^ While this study demonstrated that low-risk patients are unlikely to require ICU resources during hospitalization, it is unknown whether ICU admission might impart indirect benefits reflected in long-term neurological outcomes.

The primary objective of this study was to compare long-term neurological outcomes with ICU versus floor admission among low-risk patients with traumatic intracranial hemorrhage (tICH). The secondary objective was to compare hospital length of stay. We hypothesized that floor admission was not inferior to admission to the ICU for a favorable neurological outcome at six months. Specifically, we tested the null hypothesis that in adjusted analysis, floor admission provided no more than half the odds of a favorable neurological outcome at six months as admission to the ICU.

## METHODS

### Study design and setting

We conducted this retrospective, registry-based cohort study at a Level 1 trauma center. The study was approved by the institutional review board at the study site. At the study site, low-risk patients with tICH are generally admitted to the ICU although admission status decisions are ultimately at the discretion of the admitting trauma surgeon. Patients with tICH admitted to the ICU typically have neurological checks every hour while patients admitted to the floor typically have neurological checks every two hours or greater. All patients with tICH are evaluated by the neurosurgery service. Patients with tICH are seen for cognitive evaluation and education by speech therapists prior to hospital discharge.

### Selection of participants

Adult patients (18 years and older) with ED visits from October 1, 2008, to February 1, 2013, were identified from the hospital trauma registry using the International Classification of Diseases, 9^th^ Revision, Clinical Modification codes specific for tICH (codes 851–854). We identified low-risk patients from this cohort based on our previously derived clinical decision instrument criteria: admission Glasgow Coma Scale (GCS) score of 15, isolated head injury (defined as abbreviated injury score [AIS] less than three in all non-head body regions), age less than 65 years old, and initial head computed tomography (CT) imaging without evidence of shift or mass effect.^7^ To obtain long-term neurological outcomes, these low-risk patients were then linked to an institutional TBI database using medical record numbers and ED visit dates. Patients included in the TBI database met at least one of the following criteria that prompted neurosurgical consultation: (1) suspected TBI due to clinical history, clinical symptoms, or signs of neurological deficits on physical examination, or (2) traumatic findings on CT including any tICH. Long-term neurological outcomes were prospectively collected by trained research personnel using a standardized data collection form. Patients or their surrogates underwent a structured interview by trained research personnel at six months to assess global functioning using the Extended Glasgow Outcome Scale (GOS-E).^8^

### Methods and Measurements

Data collection followed previously published guidelines on retrospective chart review.^9^ Variables abstracted from the trauma registry included age, sex, mechanism of injury, initial ED GCS score, initial systolic blood pressure (SBP), heart rate, and respiratory rate, revised trauma score (physiological scoring system based on initial GCS, SBP, and respiratory rate),^10^ blood alcohol level, initial hematocrit, AIS score and injury severity score (ISS) (anatomical scoring system),^11^ ED disposition, hospital length of stay, in-hospital mortality, and hospital disposition. Variables abstracted from the TBI database included admission GCS score (GCS score at the time of admission), initial platelet count and international normalized ratio (INR), initial CT characteristics and prognostic score (Rotterdam CT score), 12 in-hospital neurosurgical interventions, and GOS-E score at six months.

### Outcomes

Our primary outcome measure was a dichotomized GOS-E score at six months (8 [fully recovered] versus 1–7 [not fully recovered]). The GOS-E is the most commonly used measure of global functional performance after TBI and has been recommended as the criterion standard outcome measure for TBI studies.^13^,^14^ It uses an 8-category score that is typically dichotomized between 8 and 1–7 for mild TBI to facilitate interpretation.^15^ It has excellent interrater reliability and content validity.^8^ We also conducted a sensitivity analysis adjusting the dichotomization at GOS-E 1–6 versus GOS-E 7 and 8. The secondary outcome measure was hospital length of stay (days in the hospital). Outcomes were collected independent of the knowledge of ED disposition.

### Analysis

We conducted data formatting and recoding of variables ducted using STATA 11.0 statistical software (STATA Corp, College Station, TX). The study population was characterized using descriptive statistics. Non-normal interval data were reported with medians and interquartile ranges, and proportions were presented with 95% confidence intervals (CIs). We analyzed categorical data with chi-square test or Fischer’s exact test in cases of small cell size. Continuous data were analyzed with Student’s t-test if normally distributed. We used Wilcoxon rank-sum test for nonparametric data or ordinal data. Since inherent differences likely existed between low-risk patients admitted to the ICU and those admitted to the floor we created boxplots to analyze the distribution of key independent variables (age, initial systolic blood pressure, AIS head and neck score, and Rotterdam CT score) by hospital admission location (floor versus ICU). Boxplots were also created to analyze outcome measures. Bivariate analyses on these variables and GOS-E score were also done to evaluate which variables may have influenced the GOS-E score. We then fit a logistic regression model with these variables using the dichotomized GOS-E score (8 versus <8) as the dependent variable and ED disposition as the independent variable of primary interest. A linear regression model was created to analyze hospital length of stay. To evaluate for missing data bias, we compared key characteristics between patients with and without GOS-E at six months.

## RESULTS

**Figure 1:**
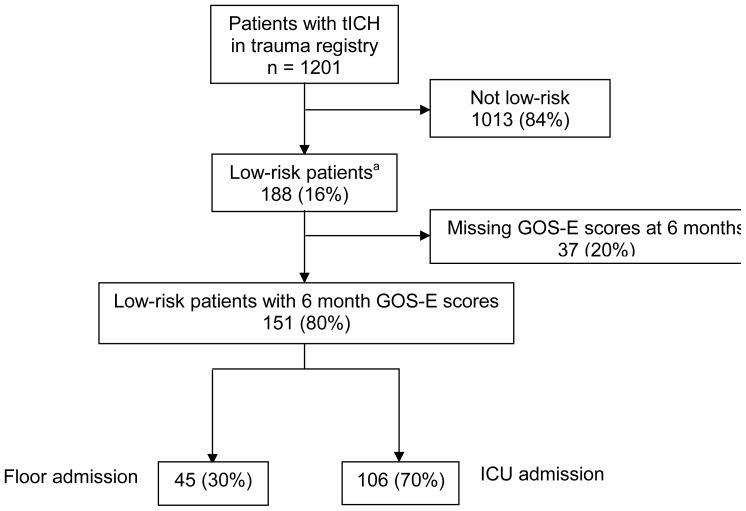
Flow of patients in the study. *tlCH*, traumatic intracranial hemorrhage; *GOS-E*, Glassgow Outcome Score Extended; *ICU*, intensive care unit ^a^ Low-risk defined as admission Glasgow Coma Scale score of 15, isolated head injury (defined as Abbreviated Injury Score less than three in all non-head body regions), age less than 65 years old, and initial head computed tomography imaging without evidence of shift or mass effect.

**Figure 2:**
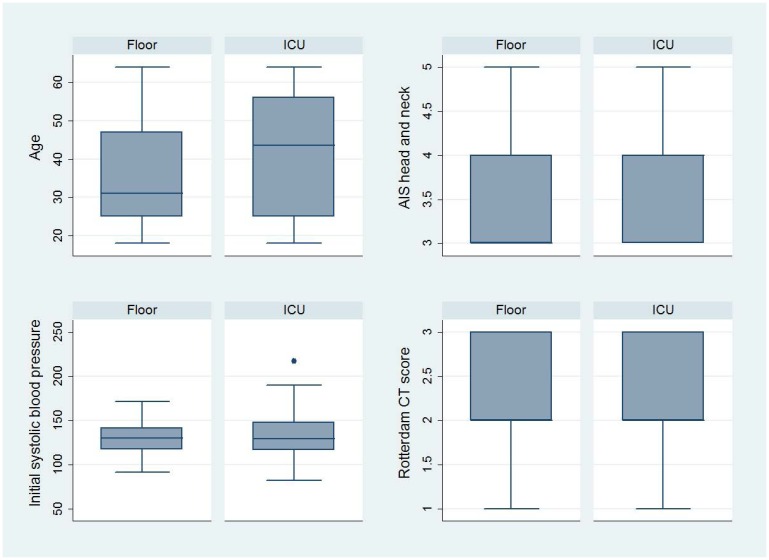
Distribution of independent variables by admission location. *ICU*, intensive care unit; *CT*, computed tomography; *AIS*, abbreviated injury score

Among 188 patients with tICH who met our inclusion criteria, 151 (80%) had complete data; 106 (70%) patients were admitted to the ICU and 45 (30%) to the floor (Figure 1). Median age in the cohort was 40 years (IQR 25–54 years) and 109 were male (72%). The most common mechanisms of injury were fall from standing (49 patients, 32%) and assault (48 patients, 32%). The most common head CT findings were subdural hematoma (56 patients, 37%) and subarachnoid hemorrhage (54 patients, 36%). One patient (admitted to ICU) required a neurosurgical intervention, which consisted of an elevation of skull fracture on hospital day 3. One patient died during hospitalization (hospital day 13) from hospital-acquired pneumonia and sepsis (see Table 1 for complete patient characteristics). Distributions of key independent variables are shown in Figure 2. The majority of patients had a GOS-E at six months of 8 (78; 52%) (Table 2). Patients with GOS-E at six months were more likely to have a higher AIS head and neck compared to patients with a missing GOS-E at six months (Table 3).

**Figure 3:**
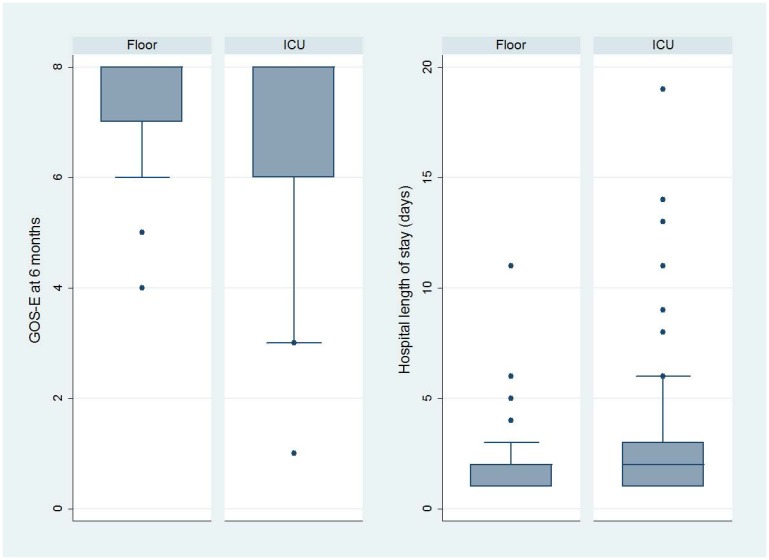
Distribution of outcome measures by admission location. *GOS-E*, extended Glassgow Outcome Score; *ICU*, intensive care unit

**Figure 4:**
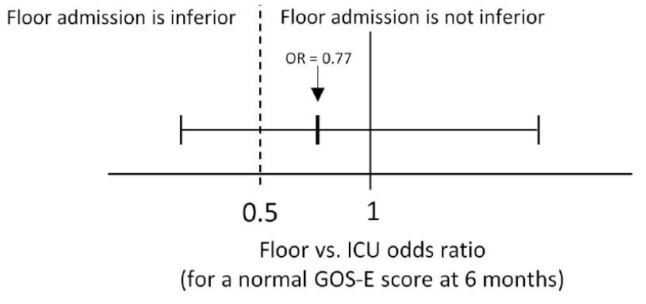
Adjusted odds ratio of floor admission on extended Glosgow Outcome Score (GOS-E) at six months (non-inferiority is not established).

Distributions of GOS-E at six months and hospital length of stay by ED disposition are shown in Figure 3. On adjusted analysis, floor admission had an odds ratio of 0.77 (95% CI [0.36–1.64]) for a GOS-E score of 8 at six months. Given our tolerance margin of an odds ratio of 0.5, we failed to reject the null hypothesis of non-inferiority (Figure 4). Only age was significantly associated with a normal GOS-E at six months (Table 4a). No variable was significant on adjusted analysis of hospital length of stay (Table 4b). In the sensitivity analysis, no variable was associated with GOS-E scores 7 and 8.

## DISCUSSION

This was a hypothesis-generating study evaluating the impact of floor and ICU admission on a clinical (neurological outcome) and system outcome (hospital length of stay) in patients with low-risk tICH. Based on the results of our study, we were unable to reject the null hypothesis of inferiority; that is, we were unable to prove that floor admission was not inferior to ICU admission by our threshold margin, because the relatively wide 95% confidence interval for the adjusted odds ratio (0.36 to 1.64) included the value 0.50. To reduce the confidence interval width in an otherwise similar study would require a larger sample size.

Our study cohort consisted of low-risk patients with tICH with the definition for “low-risk” based on a recently published derivation of a clinical decision instrument identifying patients who are unlikely to receive a critical care intervention within 48 hours of hospital admission and thus, may not require ICU admission.^7^ While these low-risk criteria narrowed the study population to a relatively homogenous, well-appearing cohort of patients with tICH, it is likely that there was still some selection bias with sicker patients more likely to be admitted to the ICU. Our analysis demonstrated that the cohort of patients admitted to the ICU were older and had a higher AIS head and neck score compared to the cohort admitted to the floor.

GOS-E scores at six months were similar to other studies evaluating patients with mild TBI.^15^,^16^ Prior studies have demonstrated that injury severity (e.g., GCS score), demographic factors including age, gender, pre-injury education and employment, as well as post-injury cognitive and social factors may influence long-term neurological outcome after TBI.^17^ To our knowledge this is the first study to evaluate the potential influence of hospital admission location on neurological outcomes. It is possible that ICU admission in low- risk patients with tICH imparts benefits such as close monitoring of neurological decline or intensive cognitive therapy that is reflected in long-term neurological function. However, the results of this study demonstrate there is no clear signal to suggest this.

## LIMITATIONS

These results should be interpreted in the context of several limitations. This study was retrospective and subject to the limitations of the chart review. While we adjusted for some potential confounders, there may be a number of unmeasured variables such as comorbidities, post-injury neurological dysfunction, and social characteristics that may have influenced outcome measures. In addition, this is a single center study, and the results may not be generalizable to other sites that have different resources or admission practices. Since these were low-risk patients with tICH, other outcome measures, such as neuropsychological impairment, psychiatric and psychological functioning and TBI-related symptoms, may be more sensitive to detect differences between management. While interrater reliability of the GOS-E has previously been shown to have acceptable agreement between trained raters, inter-rater reliability was not measured in this study.^8^ Twenty percent of patients were missing the GOS-E at six months. Patients with missing GOS-E scores may have influenced the results of the study. We did evaluate key differences between patients with and without GOS-E and found that patients with GOS-E had higher AIS head and neck scores indicating more severe injuries compared to those with missing GOS-E scores. Finally, this study was underpowered to detect small but potentially clinically important differences in neurological outcomes.

## CONCLUSION

In low-risk patients with tICH, six-month neurological outcomes were not markedly different between patients admitted to the ICU and those admitted to the floor. However, we were unable to demonstrate non-inferiority on adjusted analysis. Future work aimed at a larger, heterogeneous and prospective cohort may better evaluate the impact of ICU admission on outcomes in this patient population.

## Figures and Tables

**Table 1:** Patient characteristics by hospital admission location.

Characteristic	Floor admission (n=45)	ICU admission (n=106)
Age (years), median (IQR)	31 (25–47)	44 (25–56)
Male, n (%)	34 (76)	75 (71)
Mechanism of injury, n (%)		
Assault	13 (29)	35 (33)
Motor vehicle collision	5 (11)	11 (10)
Fall from standing	16 (36)	33 (31)
Other	11 (24)	27 (26)
Initial systolic blood pressure (mmHg), mean (SD)	129 (18)	132 (23)
Initial pulse rate (beats/min), mean (SD)	86 (17)	87 (18)
Initial respiratory rate (breaths/min), mean (SD)	17 (2)	18 (2)
Revised trauma score, mean (SD)	7.84 (0)	7.83 (0.1)
Initial hematocrit (%), mean (SD)	40.7 (5.4)	40.1 (4.8)
Blood alcohol level 80 mg/dL or more, n (%)	18 (40)	48 (45)
Initial international normalized ratio, mean (SD)	1.04 (0.08)	1.15 (1.08)
Initial platelet count (1,000s/mcL), mean (SD)	205 (65)	221 (62)
Injury Severity Score, median (IQR) *	10 (9–16)	16 (10–17)
Abbreviated Injury Scale head and neck score, median (IQR) *	3 (3–4)	4 (3–4)
Computed tomography (CT) subtypes, n (%)		
Intraparenchymal contusion	10 (22)	19 (18)
Intraparenchymal hematoma	9 (20)	24 (23)
Subdural hematoma *	10 (22)	46 (43)
Epidural hematoma	1 (2)	8 (8)
Intraventricular hemorrhage	0 (0)	2 (2)
Subarachnoid hemorrhage	20 (44)	34 (32)
Rotterdam CT score, median (IQR)	2 (2–3)	2 (2–3)
In-hospital mortality, n (%)	0 (0)	1 (0.9)
Hospital disposition, n (%)		
Discharged home	40 (89)	94 (89)
Discharged to rehabilitation facility	0 (0)	0 (0)
Discharged to skilled nursing facility	0 (0)	1 (1)
Discharged to other acute care facility	2 (4)	8 (8)
Died	0 (0)	1 (1)
Left against medical advice	1 (2)	0 (0)
Discharged to psychiatric facility or jail	2 (4)	2 (2)
Length of stay in the hospital (days), median (IQR) *	2 (1–2)	2 (1–3)
Neurosurgical intervention, n (%)	0 (0)	1 (0.9)

*ICU*, intensive care unit; *CT,* computed tomography

* p<0.05

**Table 2:** Extended Glasgow Outcome Score (GOS-E) at 6 months.

GOS-E score	Floor admission, n(%) (n=45)	ICU admission, n(%) (n=106)
8 (upper good recovery)	23 (51.1)	55 (51.9)
7 (lower good recovery)	13 (28.9)	17 (16.0)
6 (upper moderate disability)	2 (4.4)	11 (10.4)
5 (lower moderate disability)	5 (11.1)	15 (14.2)
4 (upper severe disability)	2 (4.4)	4 (3.8)
3 (lower severe disability)	0 (0)	3 (2.8)
2 (vegetative state)	0 (0)	0 (0)
1 (dead)	0 (0)	1 (0.9)

*ICU,* intensive care unit

**Table 3:** Comparison of variables for patients with and without Extended Glasgow Outcome Score (GOS-E) at 6 months.

Characteristic	With GOS-E at six months (n=151)	Without GOS-E at six months (n=37)
Age, median (IQR)	40 (25–54)	35 (21–49)
AIS head and neck score, median (IQR)*	4 (3–4)	3 (3–4)
Initial systolic blood pressure, mean (SD)	132 (21)	131 (20)
Rotterdam CT Score, median (IQR)	2 (2–3)	2 (2–3)
Admission to the ICU, n (%)	106 (70)	27 (73)

*AIS*, abbreviated injury severity; *CT*, computed tomography; *ICU*, intensive care unit

* p<0.05.

**Table 4a:** Adjusted analysis for predicting outcome measures.**

Variable	Odds ratio (95% CI)
Age	0.98 (0.95–1.00)
AIS head and neck score	0.77 (0.39–1.52)
Initial systolic blood pressure	0.99 (0.98–1.01)
Rotterdam CT Score	0.90 (0.49–1.65)
Admission to the floor	0.77 (0.36–1.64)

*GOS-E*, extended Glassgow Outcome Score; *AIS*, abbreviated injury severity; *CT*, computed tomography

* p<0.05.

** Adjusted odds ratios for predicting GOS-E of 8 at six months.

**Table 4b:** Adjusted analysis for predicting outcome measures.*

Variable	Coefficient (95% CI)
Age	0.02 (−0.01 to 0.05)
AIS head and neck score	0.64 (−0.25 to 1.52)
Initial systolic blood pressure	0.00 (−0.02 to 0.02)
Rotterdam CT Score	−0.10 (−0.91 to 0.65)
Admission to the floor	−0.28 (−4.99 to 4.99)

*AIS*, abbreviated injury severity; *CT*, computed tomography

* Adjusted coefficients for predicting hospital length of stay.
